# Anticancer Therapy-Induced Atrial Fibrillation: Electrophysiology and Related Mechanisms

**DOI:** 10.3389/fphar.2018.01058

**Published:** 2018-10-16

**Authors:** Xinyu Yang, Xinye Li, Mengchen Yuan, Chao Tian, Yihan Yang, Xiaofeng Wang, Xiaoyu Zhang, Yang Sun, Tianmai He, Songjie Han, Guang Chen, Nian Liu, Yonghong Gao, Dan Hu, Yanwei Xing, Hongcai Shang

**Affiliations:** ^1^Guang'an men Hospital, Chinese Academy of Chinese Medical Sciences, Beijing, China; ^2^Key Laboratory of Chinese Internal Medicine of the Ministry of Education, Dongzhimen Hospital Affiliated to Beijing University of Chinese Medicine, Beijing, China; ^3^Beijing University of Chinese Medicine, Beijing, China; ^4^Department of Cardiology, Beijing An Zhen Hospital of the Capital University of Medical Sciences, Beijing, China; ^5^Department of Cardiology and Cardiovascular Research Institute, Renmin Hospital of Wuhan University, Wuhan, China; ^6^Hubei Key Laboratory of Cardiology, Wuhan, China; ^7^Institute of Integration of Traditional and Western Medicine of Guangzhou Medical University, Guangzhou, China

**Keywords:** anticancer therapies, cardiotoxicity, adverse effects, atrial fibrillation, mechanisms

## Abstract

Some well-established immunotherapy, radiotherapy, postoperation, anticancer drugs such as anthracyclines, antimetabolites, human epidermal growth factor receptor 2 blockers, tyrosine kinase inhibitors, alkylating agents, checkpoint inhibitors, and angiogenesis inhibitors, are significantly linked to cardiotoxicity. Cardiotoxicity is a common complication of several cancer treatments. Some studies observed complications of cardiac arrhythmia associated with the treatment of cancer, including atrial fibrillation (AF), supraventricular arrhythmias, and cardiac repolarization abnormalities. AF increases the risk of cardiovascular morbidity and mortality; it is associated with an almost doubled risk of mortality and a nearly 5-fold increase in the risk of stroke. The occurrence of AF is also usually researched in patients with advanced cancer and those undergoing active cancer treatments. During cancer treatments, the incidence rate of AF affects the prognosis of tumor treatment and challenges the treatment strategy. The present article is mainly focused on the cardiotoxicity of cancer treatments. In our review, we discuss these anticancer therapies and how they induce AF and consequently provide information on the precaution of AF during cancer treatment.

## Introduction

Cancer is the second leading cause of mortality in America (Siegel et al., 2016). In recent years, the mortality rate for numerous malignancies has decreased due to major progress in cancer treatment. Despite such great progress, cardiotoxicity, which can affect morbidity and mortality, is often observed in numerous therapies. Some well-established anticancer drugs, such as anthracyclines, antimetabolites, human epidermal growth factor receptor 2 (HER2) blockers, tyrosine kinase inhibitors (TKIs), alkylating agents, checkpoint inhibitors, and angiogenesis inhibitors, are significantly associated with cardiotoxicity. Cardiac arrhythmia is a common complication in the treatment of cancer patients, particularly atrial fibrillation (AF) (Tamargo et al., 2015). Cardio-oncology is an emerging academic discipline designed to resolve the complicated reciprocity between cardiovascular diseases and cancer. Monitoring, early discovery, precaution, and treatment of cardiotoxicity and well-planned cancer treatment in patients with pre-existing cardiovascular diseases protect them from the possible exacerbation/persistence of cardiotoxicity and development of heart failure (HF), respectively (Albini et al., 2010; Schwartz et al., 2013; Russell et al., 2016).

**Figure 1:**
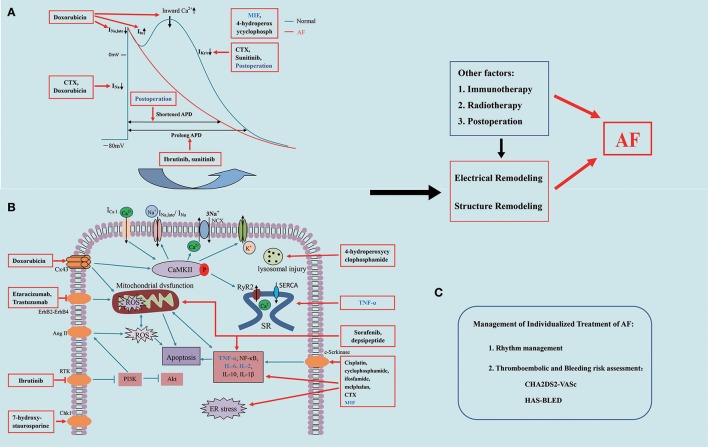
Mechanisms of anticancer therapy-induced atrial fibrillation. **(A)** Electrophysiological mechanism of anticancer drug-induced atrial fibrillation; **(B)** Signaling pathways associated with anticancer drug-induced atrial fibrillation; **(C)** Management of treatment of Atrial Fibrillation. AF, atrial fibrillation; ROS, reactive oxygen species; SR, sarcoplasmic reticular; CTX, cyclophosphamide; TNF-α, tumor necrosis factor -α; NF-κB, nuclear factor-κB; CaMKII, Ca^2+^/calmodulin dependent protein kinase II; RyR2, ryanodine receptor; ER stress, endoplasmic reticular stress; IL-2, interleukin-2; IL-6, interleukin-6; I_CaL_, L-type Ca^2+^ current; NCX, Na^+^/Ca^2+^ exchanger; I_Na_, Na^+^ channels; I_K_, K^+^ currents; SERCA, sarco/endoplasmic reticulum Ca^2+^-ATPase; Cx43/45, connexin 43/45.

One of the key issues in cancer treatment is the occurrence of AF (Farmakis et al., 2014). AF is one of the most common persistent cardiac arrhythmias, accounting for approximately one third of all patients hospitalized owing to arrhythmia (European Heart Rhythm Association et al., 2010; Fuster et al., 2011). Further, it increases the risk of cardiovascular complications, including a 3- and 5-fold increased risk of HF and stroke, respectively, and a 2-fold increased mortality rate (Ott et al., 1997; Schmitt et al., 2009; Iwasaki et al., 2011; Camm et al., 2012; Khan et al., 2013; Guo et al., 2015). AF is usually observed in patients with advanced cancer and those undergoing active cancer treatments (O'Neal et al., 2015). Anticancer drug-induced AF is common especially in poly-medicated elderly patients. The occurrence of AF is a poor prognostic element, as well as impacts therapeutic outcomes of cancer patients (Tamargo et al., 2015). The pathophysiological etiology of cancer treatment-induced AF is complicated by various cellular and biomolecular interactions, as Figure 1 indicated in the mechanisms of cancer treatment-induced AF constant chemotherapy, the immunization therapy, and cancer surgery. In our review, we discuss anticancer therapies that induce AF and what is known about their contributing mechanisms, and offer recommendations for the management of AF during treatment of cancer.

## Mechanisms of anticancer drug-induced AF

With the presence of a trigger, structural and electrical remodeling occurs, which consequently initiates AF development (Nattel, 2002; European Heart Rhythm Association et al., 2010; Fuster et al., 2011; Iwasaki et al., 2011). AF induces further structural and electrophysiological changes, which can promote its persistence (Hove-Madsen et al., 2004; Vest et al., 2005; Nattel et al., 2008; Chelu et al., 2009; Neef et al., 2010; Dobrev et al., 2011; Voigt et al., 2012, 2014). The structural changes, which can also be caused by coexisting structural cardiac diseases associated with AF along with age or by some drugs, yield a steady arrhythmogenic substrate that promotes the persistence of AF. Anticancer drugs can induce AF via all kinds of mechanisms, including electrophysiology, myocardial damage, inflammation, immune responses, apoptosis, and reactive oxygen species (ROS) production (Bracci et al., 2014; Farmakis et al., 2014).

### Electrophysiology

Changes in the myocardium can lead to abnormal electrophysiology, which can cause AF (Gupta et al., 2002). Chemotherapeutic drug-induced AF results in electrophysiological remodeling, which can include transient outward potassium current (I_to_), K^+^ current (I_Kur_), sodium channel current (I_Na_), and L-type calcium channel current (I_Ca, L_). These changes in currents involve shortening of the action potential (AP) and effective refractory period and thus maintenance of AF (Nattel et al., 2008). Considerably, the electrophysiological remodeling may also be associated with abnormal Ca^2+^ handling and the increased incidence rate of potentially pro-arrhythmic Ca^2+^ release events from the sarcoplasmic reticulum (SR) during diastole (Hove-Madsen et al., 2004; Vest et al., 2005; Chelu et al., 2009; Neef et al., 2010; Dobrev et al., 2011; Voigt et al., 2012, 2014; Xing et al., 2013). Ca^2+^/calmodulin-dependent protein kinase II (CaMKII) plays a vital part in AF by regulating cardiac-related channels and calmodulin (Neef et al., 2010; Yang et al., 2017b). Chemotherapeutic drugs can also induce CaMKII-mediated SR Ca^2+^ leakage and thus AF (Sag et al., 2011).

### Oxidative stress

According to the principle of oxidative stress, antitumor drugs, such as doxorubicin, trastuzumab, and depsipeptide, may produce superoxide anion (O^2−^), hydrogen peroxide (H_2_O_2_), and hydroxyl radicals (OH^−^) through a series of electron transfer processes under the function of various reductases and NADH dehydrogenases (Gu, 2015; Yang et al., 2017a). These free radicals can cause mitochondrial and microsomal lipid peroxidation, which can damage a variety of cells. The production of ROS is one of the main factors of cardiotoxic side effects. For example, mtDNA damage, loss of nitrous oxide (NO), changes in gene expression, and increase or decrease in autophagy are some of the causes for cardiotoxicity that all result in elevated levels of ROS (O'Neal et al., 2015; Samman Tahhan et al., 2017).

### Apoptosis

Apoptosis can eliminate aging and abnormal cells and play an important role in maintaining many cellular functions. Oxidative stress puts the body in a vulnerable state and enhances the toxic effects of pathogenic factors (Beck, 1999). It is not only related to the occurrence and development of various diseases but also has a close relationship with apoptosis (Ozaki et al., 2000). Meanwhile, the calcium ions play a major role in this process. Antitumor drugs activate the oxidative stress system of the cardiomyocytes, leading to the accumulation of ROS in the intracytoplasm. This consequently opens the ryanodine receptor on the SR of the cardiomyocytes to release a large amount of Ca^2+^ ions; thereafter, the intracellular Ca^2+^ clearance system fails, increasing the intracellular Ca^2+^ concentration (Keefe, 2001). A large number of Ca^2+^ ions causes changes in the mitochondrial membrane potential, arousing mitochondrial edema and rupture of the outer membrane, leading to the release of cytochrome c and apoptosis-induced factors, and thus promoting apoptosis of the cardiomyocytes (Gen et al., 2001).

### Inflammation

Changes in inflammation are common in the tumor therapy-induced AF (Aviles et al., 2003; Siemes et al., 2006; Erichsen et al., 2012). Inflammation, determined by elevations of the concentrations of related biomarkers, is associated with the presence or development of AF (Hernández, 2006). Furthermore, cancer-related systemic inflammation promotes and maintains AF by inducing atrial structural remodeling, such as that in tumor necrosis factor (TNF)-α, nuclear factor (NF)-κB, and macrophage migration inhibitory factor (MIF) (Guzzetti et al., 2002). NF-κB is a redox-sensitive transcription factor that causes inflammation and structural remodeling by activating TNF-α, iNOS and IL-β (Wang et al., 2018). Increased density of inflammatory mediators, such as IL-6 and high-sensitivity C-reactive protein (hs-CRP), has also been recognized as a risk factor for AF (Conway et al., 2004). Inflammation plays a significant effect in the progression of cancer, and thus AF may represent an inflammatory complication in the course of cancer treatment (Ferreira et al., 2015).

### Immune factors

Regulation of immune responses in patients with cancer and AF might be a potential target for cancer treatment. Cyclophosphamide (CTX) induces myocardial fibrosis and cardiac hypertrophy, as well as changes in the expressions of several cytokines, such as interleukin (IL)-2, IL-10, IL-6, and TNF-α, which can further facilitate the occurrence and development of AF (Liu et al., 2015). AF patients indicated a higher concentration of TNF-α and IL-6, lymphomonocyte infiltration, as well as the degree of myocardial fibrosis. Qu et al. (2009). In addition, inhibition of interleukin and TNF-α might be associated with attenuation of AF and even may be good for preventing the development of AF (Zhang et al., 2015).

## Anticancer drugs

Anticancer drug-induced adverse effects are a serious problem, as the life expectancy in cancer treatment may be decreased by the increased mortality rate owing to a series of cardiac adverse events (CAEs). Multiple widely used anticancer drugs are associated with an increasing risk of cardiotoxicity, including anthracyclines, xuoropyrimidines, alkylating agents, interferons, IL-2, taxanes, and TKIs (Table 1) (Floyd et al., 2005; Carver et al., 2007; Curigliano et al., 2010). A single anticancer drug is often employed in combination with other anticancer drugs, immunological drugs. However, the use of anticancer drugs can increase the incidence rate of AF in patients with cancer, thereby increasing the risk of mortality.

**Table 1:** AF induced by anticancer therapy.

**Classification**	**Drug classified**	**Drug**	**Incidence of AF**	**Mechanisms and actions**	**References**
Anticancer drugs	Targeted therapies	Ibrutinib, 7-hydroxy-staurosporine,	6.1%	PI3K–Akt pathway, the BTK and tec protein tyrosine kinase (TEC)	Honigberg et al., 2010; Herman et al., 2011; Burger et al., 2015; Byrd et al., 2015; Wang et al., 2015; Gertz, 2017; Shanafelt et al., 2017
	TKIs	Cetuximab, Crizotinib, Sunitinib, sorafenib	3.3%	QT interval prolongation, decrease of nitric oxide signaling, increase of endothelin-1 production, inhibited AMPK and potassium channels, enhanced accumulation of lipid, ROS production, mitochondrial disorders, and apoptosis	Lara et al., 2005; Moslehi, 2016
	Anthracycline agents	Aclacinomycin A, doxorubicin, adriamycin, 7-con-o-methylnogaril.	6.6%	Cx43/Cx45 junction channels, CaMKII, Ca^2+^ ATPase, ST segment elevated, inverted T wave, long QT intervals, ROS, mitochondrial dysfunction, and apoptosis	Kluza et al., 2004; Chu et al., 2007; Lai et al., 2011; Lau et al., 2011; Xin et al., 2011; Zhang et al., 2011; Doherty et al., 2013; Kawabata et al., 2015; Varga et al., 2015
	Alkylating agents	Cisplatin, Melphalan, CTX, 4-hydroperoxycyclophosphamide, cyclophosphamide, Ifosfamide.	15.5%	cardiomyocyte contractions, mitochondrial abnormalities, ER stress and apoptosis, ROS, and inflammation, inducing cellular sodium, calcium, potassium, ATP content, the lysosome injury	Eskilsson et al., 1988; Petrella et al., 1989; Menard et al., 1991; Tomkowski et al., 2004; Pfister et al., 2006; Richards et al., 2006; Kilickap et al., 2007; Tilleman et al., 2009; Zellos et al., 2009; Liu et al., 2015
	HER2/Neu receptor blockers	Etaracizumab, trastuzumab.	19.9%	oxidative stress, apoptosis, ErbB2-ErbB4 signaling	Kupari et al., 1990; Quezado et al., 1993
	Antimetabolites	5-Fluorouracil, leucovorin.	2.6%	the DNA synthesis, coronary spasm, myocardial ischaemia	de Forni et al., 1992; Perez-Verdia et al., 2005
	Antimicrotubule agents	Paclitaxel, Docetaxel, Gemcitabine, gemcitabinevinorelbine	9.4%	block cell division, coronary flow and left ventricular systolic pressure	Slamon et al., 1987; Keefe et al., 1993; Meydan et al., 2005
	Histone deacetylase inhibitors	Depsipeptide, Belinostat.	4.6%	No report	Bryan-Brown, 1932; Brouty-Boye et al., 1995; Alloatti et al., 1998
	Antiestrogens	tamoxifen	No report	No report	Ueda et al., 1994b
	Proteosome inhibitors	Lenalidomide, lidomide, bortezomib.		the cellular proliferation, apoptosis	Weber et al., 2003
Immunotherapy		Interleukin-2, TNF-α, MIF,	6.0%	proinflammatory cytokines, calcium homeostasis, inflammation, falling I_Ca, L_ amplitudes, and activating c-Src kinases	Thompson et al., 1994; White et al., 1994; Issac et al., 2007; Fildes et al., 2009; Rao et al., 2009; Pérez Persona et al., 2011; Guo et al., 2012a,b
Radiotherapy			No report	myocardial fibrosis	Haudek et al., 2007; Lee et al., 2007
Postoperation			10%-20%	CRP and IL-6 increased, increased K^+^ outward current, and shortened action potentials	Chung et al., 2001; Craig et al., 2001; Aviles et al., 2003; Gaudino et al., 2003; Anselmi et al., 2009; Heerdt et al., 2012; Alifano et al., 2014

*AF, atrial fibrillation; CTX, cyclophosphamide; TNF-α, tumor necrosis factor-α; ER stress, endoplasmic reticular stress; Cx43/45, connexin 43/45; BTK, bruton kinase; TEC, tec protein tyrosine kinase; HDAC, hydroxamic acid histone deacetylase; MIF, macrophage migration inhibitory factor*.

### Targeted therapies

Targeted cancer drugs are usually sorted as either micromolecules or monoclonal antibodies (Tamargo et al., 2015). They are aimed to disturb a specific signaling involved in the course of cancer progression.

Ibrutinib, a new kind of targeted anticancer drug, is a Bruton kinase inhibitor (Honigberg et al., 2010; Herman et al., 2011), which has been confirmed to be effective in some B-cell malignancies (Burger et al., 2015; Byrd et al., 2015; Treon et al., 2015; Wang et al., 2015; Gertz, 2017). In a recent meta-analysis of 20 studies surveying the occurrence of AF in patients treated with ibrutinib, the rate of AF in the ibrutinib-treated patients was distinctly higher than that in the non-ibrutinib-treated patients and the age-matched normal subjects (Leong et al., 2016; Yun et al., 2017). The mechanism by which ibrutinib induced cardiotoxicity likely involved the reduction of the PI3K signaling in the heart, which may increase the susceptibility to AF. McMullen et al. revealed that ibrutinib was able to suppress the PI3K-Akt signaling in an isolated rat myocardial cell (Pretorius et al., 2010; McMullen et al., 2014). In another study, ibrutinib triggered aberrant APs in isolated mouse and rabbit myocardial cells, and the defects were quickly reversed by adding PI3K to the pipette (Yang et al., 2015). These results indicate that ibrutinib causes AF by inhibiting the PI3K-Akt pathway in the heart. A previous study has shown that patients treated with ibrutinib without a history of AF had an incidence rate of AF of 6.1% (Shanafelt et al., 2017). Some patients even stopped treatment with ibrutinib owing to the occurrence of AF (Byrd et al., 2014). Thus, it is necessary to conduct further studies on the mechanism of ibrutinib-induced cardiotoxicity.

There are other drugs that can cause AF during cancer treatment. With the recent use of checkpoint inhibitors, the clinical outcomes of patients with tumors, such as metastatic melanomas and renal, lung, and bladder tumors, have dramatically improved (Ryder et al., 2014; Wolchok, 2015; Yu et al., 2015; Lee et al., 2016; Moslehi, 2016). Specifically, 7-hydroxy-staurosporine (UCN-01) is a new type of an antitumor drug. A previous phase I trial aimed to ascertain the safety and the pharmacokinetics of ascending doses of cisplatin combined with UCN-01 in patients with malignant tumors (Lara et al., 2005). Ten patients were enrolled, and treatment was halted at dose level 2 owing to dose-limiting toxicity (DLT) grade 3 AF in one patient.

### TKIs

Tyrosine kinase inhibitors are significant targets for cancer treatment because they play an important role in the regulation of growth factor signaling (Guglin et al., 2009). In the chronic myelogenous leukemia, the BCR-Abl kinase is a tyrosine kinase target. Several kinds of TKIs containing nilotinib, erlotinib, dasatinib, and imatinib have targeted the kinase. These anticancer agents had been reported to induce AF, thromboembolism, and pulmonary hypertension. Some studies have reported that cetuximab, sunitinib, and alemtuzumab were linked to AF in the one case report each (Lenihan et al., 2004; Pfister et al., 2006; Mego et al., 2007). Rituximab is related with numerous reactions containing cardiac arrhythmias, such as AF and ventricular tachycardia (VT), reversible after the discontinuation of medication (Coiffier et al., 2002; Arai et al., 2005).

Sunitinib is a kind of drug which can selectively target many kinds of receptor tyrosine kinases. It works by blocking blood and nutrients needed for tumor growth. In patients with metastatic gastrointestinal stromal tumor, sunitinib treatment is reported to induce left ventricle (LV) contractile dysfunction (Chu et al., 2007). Multiple target points, such as reduced myocardial cell activity, inhibited AMPK and K^+^ channels, and enhanced accumulation of lipids, are also reported (Doherty et al., 2013). These underlying mechanisms of sorafenib-induced cardiotoxicity are linked with LV contractile dysfunction, ROS production, mitochondrial disorders, and apoptosis in the myocardial cell (Will et al., 2008; Duran et al., 2014; Kawabata et al., 2015).

### Anthracyclines

Anthracyclines antibiotic inhibits cell growth and restrains the fleetly increasing cancer cells (Guglin et al., 2009). They are well known for the associated cardiotoxicity. There are many mechanisms underlying the cardiotoxicity associated with doxorubicin use (Gorelik et al., 2003). Dog and sheep models treated with doxorubicin showed some anomalous electrocardiogram findings, including ST segment elevation, T wave inversion, QT interval prolongation, and cardiac arrhythmia (Lau et al., 2011; Xin et al., 2011). A previous study has recognized that doxorubicin can down-regulate the expression of the Cx43/Cx45 junction, resulting in cardiac dysfunction and LV remodeling (Zhang et al., 2011). Doxorubicin-induced mitochondrial dysfunction (Varga et al., 2015), ROS production (Kluza et al., 2004), and apoptosis were observed in the cardiomyocytes (Lai et al., 2011). Doxorubicin suppresses the expression of the SR Ca^2+^ ATPase, impairing Ca^2+^ regulation and consequently cardiac function (Arai et al., 1998). Doxorubicin use can also lead to CaMKII-mediated Ca^2+^ leakage from the SR, which can destroy the intracell Ca^2+^ steady state and increase the incidence rate of AF (Bracci et al., 2014). CaMKII acts a crucial part in the occurrence and development of AF via regulating Ca^2+^-related proteins and cardiac ion channels, such as L-type Ca^2+^ currents, Na^+^ currents, and late Na^+^ currents. In addition, CaMKII inhibition can decrease the cardiotoxicity induced by doxorubicin (Bracci et al., 2014), which demonstrates the underlying CaMKII regulation regarded as a policy for alleviating anticancer drug-induced AF.

In a phase I clinical trial (Woolley et al., 1982), 22 patients with cancer were administered with the new anthracycline aclacinomycin A, and one patient developed transient AF. Conversely, 7-con-O-methylnogaril was also a novel chemotherapeutic drug used in clinical trials (Dorr et al., 1986). Twenty-four patients received this drug, and one patient developed cardiotoxicity (transient AF). A clinical study recorded paroxysmal AF in 6.9% of 393 patients during the first course of doxorubicin chemotherapy (Numico et al., 2002). Other studies have also reported similar findings (Montella et al., 2005; Kilickap et al., 2007; Lebedinsky et al., 2011). In summary, the cardiotoxicity induced by anthracyclines has been well researched, as well as association of anthracyclines with AF appears to be closely connected.

### Alkylating agents

Alkylating agents (e.g., cisplatin, CTX, ifosfamide, and melphalan) can also cause AF (Eskilsson et al., 1988; Petrella et al., 1989; Menard et al., 1991; Moreau et al., 1999; Ifran et al., 2005; Pfister et al., 2006). They are normally used for the treatment of slow-growing cancers. Cisplatin has been employed extensively for locoregional perfusion in thoracic malignancies. A great number of previous studies have indicated that cisplatin-induced cardiotoxicity may result in LV dysfunction, restrained myocardial contractions (Ma et al., 2010), mitochondrial dysfunction (Pfister et al., 2006), strengthened endoplasmic reticular stress, cell apoptosis (Honigberg et al., 2010), ROS production, and inflammation (Ma et al., 2010). Cardiotoxicity is induced by cisplatin via upregulation of TNF-α and NF-κB (Albini et al., 2010). The administration of 4-hydroperoxycyclophosphamide to the cardiomyocytes stimulates cytotoxicity by inducing cellular Na^+^, Ca^2+^, and K^+^ activation, ATP content (Feliz et al., 2011), and lysosomal injury (Sudharsan et al., 2006). Finally, CTX use leads to myocardial hypertrophy, myocardial fibrosis, and changes in the expressions of some cytokines, such as IL-1β, TNF-α, and IL-10, which are likely to promote AF development (Liu et al., 2015).

Among patients with adenocarcinoma of the lung and pericardial tamponade who received cisplatin perfusion, 19% showed AF (Tomkowski et al., 2004; Richards et al., 2006; Tilleman et al., 2009; Zellos et al., 2009). In a recent clinic trial, carboplatin combination therapy induced AF in one of 32 patients (Illiano et al., 2000). Using high doses of CTX and ifosfamide increases the risk of paroxysmal supraventricular tachycardia and paroxysmal AF (Kupari et al., 1990; Quezado et al., 1993). In 11% of patients who underwent bone marrow transplant with high-dose melphalan treatment, AF was observed (Olivieri et al., 1998; Moreau et al., 1999; Abidi et al., 2012).

### HER2 blockers

A previous experimental study has confirmed that trastuzumab-induced cardiotoxicity was associated with enhanced myocardial ROS production, apoptosis, and changes in the ultrastructure (Elzarrad et al., 2013). Another study indicated that trastuzumab use correlated with LV contractile dysfunction was regulated by the combination with the HER2 protein, accordingly interdicting the ErbB2-ErbB4 signaling channel (Jones et al., 2009). Approximately 19.9% of female patients discontinued trastuzumab treatment because of AF development. Another blocker used in patients with previously untreated metastatic melanoma was etaracizumab, an IgG1 humanized monoclonal antibody against the avb3 integrin. After treatment with etaracizumab, 9% of patients had AF (Hersey et al., 2010).

### Antimetabolites

Antimetabolites are specifically bound to metabolites in the body and thus affect or antagonize metabolic functions. They have a chemical structure similar to that of nucleic acids or protein metabolites in the body. They play an antitumor role by interfering with DNA synthesis. It has been reported that the incidence rate of cardiotoxicity reached up to 2-4% in patients with cancer receiving antimetabolites, such as 5-fluorouracil (FU) or other analogs (Berliner et al., 1990; de Forni et al., 1992; Frickhofen et al., 2002; Perez-Verdia et al., 2005; Saif et al., 2009). Particularly, 5-FU is a synthetic pyrimidine antimetabolite, which acts as a cell growth inhibitor to malignant lesions; however, the cardiotoxicity associated with this has only been investigated in some clinical studies. In a previous case report, AF was found in a 60-year-old male patient within the first 24 h after receiving 5-FU treatment (Aziz et al., 1998). Meydan et al. (2005) surveyed the incidence rate of cardiotoxicity associated with high-dose leucovorin combined with 5-FU continuous infusion, and the patients underwent long-term follow-ups. They found that nine of 231 patients who were administered with high-dose leucovorin combined with 5-FU developed cardiotoxic events, revealing an overall occurrence rate of 3.9%. Myocardial ischemia appears to dominate 5-FU-induced cardiotoxicity; however, many cardiac arrhythmias appear in ischemia-reperfusion injuries as ventricular arrhythmias, AF, etc. (Slamon et al., 1987; Keefe et al., 1993; Hrovatin et al., 2006).

### Antimicrotubule drugs

Tubulin, an antimicrotubule drug, plays a significant part in intracellular transportation, cell mitosis, and signal transduction. Paclitaxel is a kind of microtubulin polymerization agent, and has become an important treatment for lung, breast, and ovarian cancer. However, as with other antitumor drugs, the side effects and the emergence of resistance after administration limit the clinical use of microtubulin inhibitors. Paclitaxel cardiotoxicity can lead to AF, VT, ventricular fibrillation (VF), and even sudden mortality (Arbuck et al., 1993), and gradually increases with the time and dosage of the drug use (Brouty-Boye et al., 1995). There were 90 patients who were administered with paclitaxel as the second-line chemotherapeutic drug, and considering the cardiovascular events that occurred, the incidence rate of AF was 1%. In the perfused heart of guinea pigs, paclitaxel caused an abnormal conduction and consequently decreased coronary blood flow and LV systolic pressure (Alloatti et al., 1998). Meanwhile, in a study on frogs and rabbits, taxanes slowed the heart rate and generated auriculo-ventricular block, thereby leading to asystole (Bryan-Brown, 1932). In a randomized phase 3 trial, two studies employed gemcitabine and gemcitabine vinorelbine (Gridelli et al., 2001). Forty-nine patients participated in each group; in the gemcitabine vinorelbine combination group, four patients developed serious cardiotoxicity complications accompanied with atrial flutter or AF.

### Histone deacetylase inhibitors

Depsipeptide is a histone deacetylase inhibitor, which can regulate gene expression and adjust cell cycle arrest and cell apoptosis. Studies have shown that it could validate cytotoxicity suppressing the human tumor cell lines (Ueda et al., 1994a,b). Based on a large amount of pre-clinical data, depsipeptide is likely to have conspicuous cardiotoxicity. There were 88 patients who received depsipeptide treatment in a clinical study (Sandor et al., 2002), and the DLT involved grade-4 arrhythmia in one patient (AF). Stadler et al. (2006) investigated some patients with refractory renal cell carcinoma, who participated in a phase II study. One patient developed grade 3 AF. Belinostat is also a new hydroxamic acid histone deacetylase inhibitor with potent antiproliferative activities (Plumb et al., 2003). Conversely, Steele et al. investigated (Steele et al., 2008) 46 patients who received treatment of belinostat, and the DLT involved grade 3 AF.

### Antiestrogens

Some studies have shown that estrogen plays an important role in the occurrence and development of breast cancer. Approximately two-thirds of breast cancer cells contain a certain amount of estrogen receptors (Robertson et al., 1996). Tamoxifen is one of the most common estrogens blockers; it is often used to treat advanced breast and ovarian cancers. The effectiveness rate of clinical breast cancer treatment is generally 30%. There were 5,408 women who underwent hysterectomy and were distributed among the tamoxifen and placebo groups (Veronesi et al., 2007). AF occurred more often in the patients who received tamoxifen treatment.

### Proteasome inhibitors

Proteasomes are a colossal protein composite existing in the cells, which can degrade other proteins, block cellular proliferation, and induce apoptosis in the tumor cells, particularly in multiple myelomas (MMs). The conditions of patients with MMs have changed prominently during the past few decades with the introduction of new drugs, such as thalidomide, bortezomib, and lenalidomide (Kumar et al., 2008). Dexamethasone is commonly used in combination with these drugs to treat cancer. The combination of lenalidomide and dexamethasone is perceived as a special treatment option in these patients. A critical aspect in the clinic application of lenalidomide is active monitoring for CAEs (Zangari et al., 2001, 2009; Neben et al., 2002; Weber et al., 2003; Zonder et al., 2006; Palumbo et al., 2008; Klein et al., 2009). Although these drugs have changed the therapeutic effect on MMs, substantially improving patient outcomes, their use easily induces cardiotoxicity. The primary CAE associated with lenalidomide use is AF, particularly when it is employed for a long period (Pérez Persona et al., 2011).

## Immunotherapy

Interleukin refers to a lymphokine that interacts between leukocytes and immune cells, and is a cytokine of the same type as the hematopoietic growth factor, coordinating and interacting with each other to complete hematopoiesis and immune regulation. IL-2 can mediate tumor treatment in patients with renal cell carcinoma and metastatic melanoma (White et al., 1994). Considering the Food and Drug Administration approval, patients with renal cell carcinoma are treated with high-dose recombinant IL-2; however, its use has a high incidence rate of cardiotoxicity. ILs are found in various cancer-induced cardiotoxicities, including hypertension, HF, and AF (Aoyagi and Matsui, 2011; Guo et al., 2012a,b), revealing elevated concentrations of pro-inflammatory cytokines, such as IL-6 and IL-2 (Fildes et al., 2009; Guo et al., 2012a). In affected patients, IL-6 was highly expressed, which was closely associated with the AF duration (Issac et al., 2007). There were 199 patients who received 310 treatment courses. Cardiac arrhythmia occurred in 6% of the patients, with 11 of these patients retreated and two who showed AF recrudescence. Thompson et al. (1994) conducted a phase lb clinical test in sick patients receiving IL-2 combined with lymphokine-activated killer cell treatment for metastatic renal cell carcinoma; 18 patients were administered with IL-2, and AF occurred at a dose of 4.9 mg/kg.

Macrophage MIF plays an important role in the inflammatory pathways and is associated with the occurrence of many cancer phenotypes (O'Reilly et al., 2016). Some studies also found that inhibiting the function of MIF could significantly reduce the growth of cancer *in vitro* or *in vivo* systems, such as bladder cancer, lung cancer, and colon cancer (Choudhary et al., 2013; Kindt et al., 2013; Ioannou et al., 2014; Mawhinney et al., 2014; Varinelli et al., 2015). MIF also plays a crucial role in the pathophysiology of cardiovascular diseases (van der Vorst et al., 2015). A previous study also showed that MIF was related to electrical remodeling with AF, probably through falling I_Ca, L_ amplitudes and activating c-Src kinases in the atrial myocytes (Rao et al., 2009). In summary, these studies highlight the importance of controlling MIF expression in preventing atrial electrical remodeling in patients with cancer.

TNF-α is deemed as a primary moderator of immune responses and a vital participator in the cytokines (Balkwill, 2009). It is generally involved in the upregulation of all kinds of chemotherapeutic drug-induced cardiotoxicity (Karayiannakis et al., 2001; Balkwill, 2002; Szlosarek and Balkwill, 2003) and can affect patients' prognosis (Balkwill, 2006). A previous study deemed TNF-α as a pivotal regulator in colon cancer progression and proved that interdicting TNF-α in a mice model can lessen colonitis-related carcinoma of the colon (Popivanova et al., 2008). TNF-α yields apoptosis of various cells (Haudek et al., 2007) and enhances PV arrhythmogenicity and Ca^2+^ homeostasis maladjustment, thus resulting in the occurrence of AF (Lee et al., 2007).

## Radiotherapy

With the application of radiotherapy technology, treatment of malignant tumors and some benign diseases by ionizing radiation has significantly reduced mortality. The cardiovascular toxicity of chest radiotherapy increases cardiovascular mortality, partially offsetting the improvement in survival rate for chest radiotherapy (Qi and Zhang, 2015). The main manifestations of radiation therapy for cardiotoxicity are ischemic heart disease, HF, and AF. Among patients who were treated for breast cancer between 1980 and 2000, the cardiotoxicity risk was highest in the patients treated with left breast radiotherapy (Hooning et al., 2007). Significant myocardial fibrosis is very common in the radiotherapy-induced cardiotoxicity (Jaworski et al., 2013). Although there is a lack of clear evidence, it is assumed that radiotherapy could also provoke AF by the occurrence and development of myocardial fibrosis and HF (Mery et al., 2017).

## Postoperation

In recent years, the incidence of AF after thoracic surgery has increased, and postoperative AF (POAF) in cancer is closely associated with inflammation and sympathetic activation (Mc Cormack et al., 2014). AF is a common complication of postoperative lung cancer. Approximately 10–20% of patients develop AF, which occurs about 2–3 days after surgery (Vaporciyan et al., 2004; Roselli et al., 2005; Gómez-Caro et al., 2006). Meanwhile, many reports found POAF occurrence and proinflammatory cytokines activated (Bruins et al., 1997; Chung et al., 2001; Aviles et al., 2003; Gaudino et al., 2003; Anselmi et al., 2009). In some studies, for example, CRP and IL-6 increased in the lung cancer patients after surgery (Craig et al., 2001; Alifano et al., 2014). Moreover, there are also some studies that show atrial KCNE1 (potassium channel subunit) down-regulation, which indicated an increased outward current and shortened action potentials in POAF (Heerdt et al., 2012). In addition, POAF happened in 12.6% of the colorectal cancer patients receiving elective colectomy, and also occurred in 9.2% of the esophageal cancer patients after esophagectomy (Siu et al., 2005; Ojima et al., 2014). AF may be regulated by sympathovagal nerve injury following surgical trauma (Amar et al., 1996; Ma et al., 2006), which plays a crucial effect on the occurrence of AF.

## Prevention and management

In view of the lack of evidence, there is no specific guideline for the treatment of AF in patients with malignant tumors. The prevention and treatment of AF are based on the current guidelines for the practical management of patients with and without cancer (Chelu et al., 2009; Anderson et al., 2013). The therapeutic management should be individualized, and the decisions regarding anti-arrhythmic drugs or instrumental treatments (Priori et al., 2015) should consider the contending risks of cancer, cardiac-related life expectancy, living quality, and risks of complications. The management of antitumor therapy-induced AF mainly has two aspects: (1) Rhythm control can prevent AF and ameliorate optimal rate control symptoms in patients who still have symptoms (Ferrari et al., 2016). The original method used in managing AF requires the usual decisions regarding rhythm management, particularly in terms of antithrombotic therapy for stroke prevention. So, in the future, more personalized rhythm control therapies could help ameliorate the therapeutic effect and security of therapy. (2) Some patients with cancers in the blood are prone to coagulation defects resulting in bleeding, which may be contraindicated for antithrombotic therapy. Some patients with cancer, such as lung cancer and primary liver cancer, have an increased risk of thromboembolism and therefore, need to be evaluated using risk assessment tools. An assessment tool for antithrombotic therapy in cancer-induced AF, according to cancer features, and established thromboembolic and bleeding risk assessment tools, such as the CHA2DS2-VASc and HAS-BLED scores, are used (Lee, 2005; Hu et al., 2013). Hence, the decision regarding the initiation of antithrombotic treatment in patients with cancer has to be austerely individualized, weighing modestly the benefits against the risks based on the characteristics of every specific patient.

## Conclusions

In this review, the mechanisms of some chemotherapeutic drugs, post-surgery, radiation therapy, and cancer system immunity in inducing AF were summarized on the basis of existing data. We hope to attract more attention of cardiologists to this problem. As anticancer therapy-induced AF usually occurs in cancer centers, clinically relevant data on treatment, risk of embolic events, persistence period, and particularly ischemic strokes are not available in the literature. Moreover, the development of AF may impact the therapeutic effects of people with cancer. Therefore, it is necessary to understand the potential mechanism of AF occurrence in people with cancer, which can help increase the effectiveness of cancer treatments. As the field of oncocardiology expands, cardiac oncologists need to know the fundamental electrophysiology principles and management so as to offer proper care for people with cancer.

## Author contributions

YX and HS confirmed the article theme. YY, TH, NL, and SH looked for related articles. CT, MY, XZ, YS, GC, and YG collated all related articles. XY wrote the manuscript. XL, XW, and DH modified this manuscript. All authors commented on the manuscript.

### Conflict of interest statement

The authors declare that the research was conducted in the absence of any commercial or financial relationships that could be construed as a potential conflict of interest.
